# GHRH, PRP-PACAP and GHRHR Target Sequencing via an Ion Torrent Personal Genome Machine Reveals an Association with Growth in Orange-Spotted Grouper (*Epinephelus coioides*)

**DOI:** 10.3390/ijms161125940

**Published:** 2015-11-02

**Authors:** Liang Guo, Junhong Xia, Sen Yang, Mingming Li, Xinxin You, Zining Meng, Haoran Lin

**Affiliations:** 1 State Key Laboratory of Biocontrol, Institute of Aquatic Economic Animals, and the Guangdong Province Key Laboratory for Aquatic Economic Animals, Sun Yat-Sen University, Guangzhou 510275, China; zsdxgl@163.com (L.G.); xiajunh3@mail.sysu.edu.cn (J.X.); yangsen04@163.com (S.Y.); yoursliming11@163.com (M.L.); lsslhr@mail.sysu.edu.cn (H.L.); 2 Guangdong Provincial Key Lab of Molecular Breeding in Marine Economic Animals, BGI, Shenzhen 518083, China; youxinxin@genomics.cn

**Keywords:** GHRH, PGM, association analysis, growth, orange-spotted grouper

## Abstract

Growth hormone-releasing hormone (GHRH) and the receptor, GHRHR, constitute important components of the hypothalamus-pituitary growth axis and act on the downstream growth hormone (GH). PACAP-related peptide/pituitary adenylate cyclase activating polypeptide (PRP-PACAP*)* is a paralog of GHRH. These genes all play key roles in development and growth patterns. To improve the quality of cultured fish strains, natural genetic variation must be examined and understood. A mixed linear model has been widely used in association mapping, taking the population structures and pairwise kinship patterns into consideration. In this study, a mass cross population of orange-spotted grouper (*Epinephelus coioides*) was examined. These candidate genes were found to harbor low nucleotide diversity (θ_w_ from 0.00154 to 0.00388) and linkage disequilibrium levels (delay of 50% within 2 kbp). Association mapping was employed, and two single-nucleotide polymorphisms (KR269823.1:g.475A>C and KR269823.1:g.2143T>C) were found to be associated with growth (false discovery rate *Q* < 0.05), explaining 9.0%–17.0% of the phenotypic variance. The association of KR269823.1:g.2143T>C was also found via haplotype-based association (*p* < 0.05). The identified associations offer new insights into gene functions, and the associated single-nucleotide polymorphisms (SNPs) may be used for breeding purposes.

## 1. Introduction

Orange-spotted grouper (*Epinephelus coioides*) belongs to the Serranidae family and is broadly found in tropical and subtropical open oceans and seas [1]. It serves as the top predator in calm, shallow waters. In Southeast Asia, it also represents one of the most popular forms of seafood and has considerable economic value. The orange-spotted grouper has been treated as a model species used in the determination of genetic and physiological features of grouper. Their robustness in overcrowded conditions, rapid growth at high temperatures and insufficient supplies of wild caught fish render it one of the most important aquaculture species [2]. Although artificial propagation procedures have been applied in this species, fry in aquaculture production are mainly captured from the wild. The unreliable quality of fry collected from highly diversified environments might contain high genetic diversity and could not meet the demand in practice. The pressure for reliable stocks calls for the development of high quality breeds. No effective breeding program that improves the genetic quality of this species currently exists, though sporadic reports have been published on the issue. You *et al.* [3] constructed the first high-density genetic linkage map, and Wei *et al.* [4] and Huang *et al.* [5] examined the association between the *Leptin* gene and growth patterns.

Generally speaking, linkage mapping based on families has been used as a standard approach for the genetic improvement of aquaculture species [6]. However, this species is a protogynous hermaphrodite with a relatively long reproduction cycle, which hinders the establishment of families, and the species is also characterized by a short culture history and by a relatively small commercial breeding population. Thus, association mapping serves as an alternative way to locate genes underlying growth and development patterns [7]. Association mapping methods are preferable in that no segregating population needs to be constructed and mapping resolutions used can be higher relative to those used in family-based linkage mapping. Mixed linear model (MLM) approaches have been introduced as improved means of simultaneously accounting for population structures and unequal relatedness between individuals relative to general linear models, which were used by Wei *et al.* [4] and Huang *et al.* [5]. MLM-based methods have been widely employed to study human diseases [8] and plant and animal breeding patterns [9,10], typically through genome-wide association studies (GWAS) aided by highly effective genotype technologies. Meanwhile, approaches focusing on candidate genes have also been widely applied, mainly in consideration of cost and resolution levels [11,12]. Association mapping can make use of crossovers in history, and the linkage disequilibrium (LD) levels determine mapping resolutions and costs to a certain extent. Typically, LD delays with selfing species distance are long, and haplotype blocks can be larger (e.g., *Arabidopsis thaliana* [13]). Thus, association mapping in such species requires the use of fewer markers to cover an entire genome or target region. For outcrossing species (e.g., the orange-spotted grouper), LD levels may be low, and resolution levels may be higher. Thus, more intensive markers are needed to dissect genomic sequence functions. Though widely used in human disease dissection and for plant genetic improvements, MLM-based association mapping has been less frequently applied to the study of marine fish. Recently, this method has been used to map disease resistance traits in rainbow trout [14] and to map growth in Asian seabass [15].

Marker-assisted selection (MAS) methods always focus on genes that control important phenotypes in the agricultural sector. The growth hormone-releasing hormone (GHRH), PACAP-related peptide/pituitary adenylate cyclase activating polypeptide (PRP-PACAP), and GHRH receptor (GHRHR) genes have been widely studied in the field of physiology (including in the orange-spotted grouper [16]) and are supposed to control growth hormone (GH) release. The ancestral PRP-PACAP gene duplicates into two copies and independently mutates into GHRH and PRP-PACAP, and two copies of PRP-PACAP are produced via actinopterygian-specific genome duplication [17]. The primary function of mammalian GHRH is to stimulate *GH* synthesis and secretion from the pituitary by binding to its receptor, GHRHR [16]. PRP and PACAP are encoded within the same precursor protein, PRP is reported to regulate reproduction [18] and PACAP is involved in circadian rhythms, learning and memory processes, and in neurite outgrowth, cell differentiation, and proliferation [19]. Mutations in GHRH [20], GHRHR [21], and PRP-PACAP [22] genes have been reported to influence growth patterns, and reports have largely focused on domestic animals.

In recent decades, sequencing technologies have altered research methods. Several markers have been used to dissect genomes, including those of restriction fragment length polymorphism (AFLP), random amplified polymorphic DNA (RAPD), simple sequence repeat (SSR), and single-nucleotide polymorphism (SNP). Since 2005, next-generation sequencing approaches have accelerated research on genomic sequences. SNPs markers have grown popular owing to the advantages of high-density in genome and to their ease of development with the assistance of robust high-throughput sequencing tools. Ion Torrent Personal Genome Machine (PGM) is one of these high-throughput sequencing platforms and was released at the end of 2010 [23]. With no requirements for fluorescence and camera scanning, it is more efficient, cheaper, and smaller in size [24]. It is notable that its limited throughput renders the platform suitable for use in small genomic or target sequencing, and its three differently priced sequencing-chip reagents render it more flexible, though PGM is less accurate when determining homopolymers [25]. Since its release, the platform has been widely used for medical diagnoses of target [26] and bacteria genome sequencing [27].

Based on limited research previously conducted on orange-spotted grouper population and genetic attributes, the GHRH, GHRHR and PRP-PACAP genes were used to examine nucleotide diversity and LD levels at the gene scale. MLM-based association mapping was performed to dissect growth traits in individuals of a mass cross breeding population. This basic study on these four genes will provide information on population characteristics while promoting the selection breeding of this species.

## 2. Results

### 2.1. The Phenotype and Genotype

The morphometric characteristics of 159 individuals were examined in this study. The maximum value was roughly two to three times that of the minimum value for each trait. Among the 12 traits examined, eight traits were normally distributed (*p*-value > 0.05) among the sampled individuals with four exceptions: caudal peduncle depth (CPD), snout length (SNL), eyeball diameter (ED) and interorbital distance (ID). The eleven recorded traits were significantly positive correlated (*p*-value < 0.01). Condition factor (K) showed highly significant negative correlations (*p*-value < 0.01) with total length (TL) and standard length (SL), and was significant negative correlated (*p*-value < 0.05) with head length (HL), caudal peduncle length (CPL), and interorbital distance (ID).

**Figure 1:**
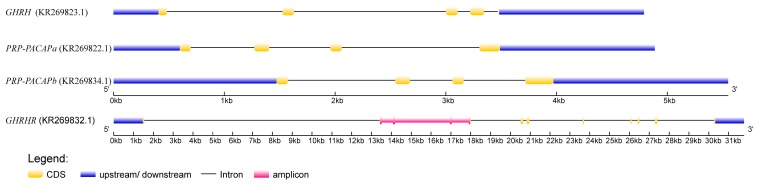
Illustration of the structure of the candidate genes. Fragments of the growth hormone-releasing hormone (GHRH) and PACAP-related peptide/pituitary adenylate cyclase activating polypeptide a (PRP-PACAPa) and b (PRP-PACAPb) genes included entire genes, and fragment from the GHRH receptor (GHRHR) gene is denoted by a pink line. Sequences of these four fragments were submitted to NCBI, and accession numbers were KR269823.1, KR269822.1, KR269834.1 and KR269832.1, respectively.

**Figure 2:**
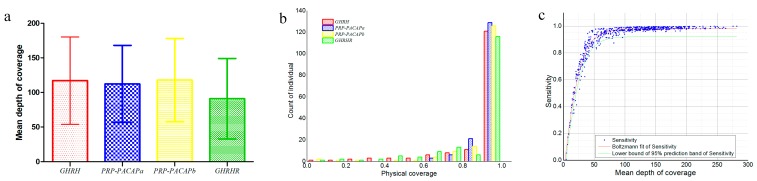
(**a**) Summary of coverage depths of the four fragments (mean ± SD); (**b**) Summary of amplicon physical coverage (sensitivity) levels. The effective site was considered when the coverage depth was less than 10-fold; and (**c**) Plot of the relationship between mean coverage depth and sensitivity levels. The regression curve was fit with the Boltzmann function, and the lower boundary of the 95% prediction band of sensitivity is shown. Data were collected from all of the amplicons.

The structure of four targeted fragments is illustrated in Figure 1. The GHRH gene was found to include five coding regions, and genes PRP-PACAPa and PRP-PACAPb were both found to include four coding regions. The GHRHR includes 13 coding regions. In this study, each of the three fragments (KR269823.1, KR269822.1, and KR269834.1) included all of the coding regions (GHRH, PRP-PACAPa and PRP-PACAPb, respectively). Meanwhile, the KR269832.1 fragment just contains regions of the GHRHR gene’s second to fifth exons. The effective lengths of the amplicons were recorded at 4756, 4852, 5550 and 4280 bp, respectively, and GC contexts were recorded at 36.88%, 41.53%, 40.70% and 40.70%, respectively. The amplicons were subjected to sequencing on the PGM platform. The average read length was found to be 208 bp without excessively short reads (length < 50 bp). Coverage depth and physical coverage (sensitivity) levels were used to assess sequencing performance levels, and sites with depths of at least 10-fold were deemed effective sites in calculating physical coverage levels. In summary, mean coverage depth and sensitivity levels were found to be 110 ± 60-fold and 0.90 ± 0.16 (mean ± SD), respectively. Average coverage depths for each fragment are listed in Figure 2a. In total, 85.8% amplicons achieved sensitivity levels of at least 80% (Figure 2b). The two individual and fragment factors significantly influenced mean coverage depth and sensitivity levels of each amplicon (Table 1). Clearly, more variance was introduced from the fragment than from the individual. The mean variance of the mean coverage depth from the fragment was found to be 2.61 times that from the individual, and the mean variance of sensitivity from the fragment was recorded at 2.21 times of that from the individual.

**Table 1:** Two-way analysis of variance on individual and fragment effects on mean coverage depths and sensitivity levels for each amplicon.

Source	Sum of Squares	Degrees of Freedom	Mean Square	F	*p*-Value
Mean coverage depth					
Individual	1,543,230	158	9767.28	6.786137	1.8 × 10^−59^
Fragment	76,483	3	25,494.44	17.71309	6.5 × 10^−11^
Error	682,227	474	1439.30		
Total	2,301,941	635			
Sensitivity					
Individual	7.7933	158	0.0493	2.794009	1.0 × 10^−17^
Fragment	0.3277	3	0.1092	6.187525	4.0 × 10^−4^
Error	8.3679	474	0.0177		
Total	16.4889	635			

The relationship between sensitivity and the mean coverage depth was fitted to a non-linear regression model. A correlation coefficient (*r*^2^) of 0.94 was found, denoting that the relationship is well depicted by the model and that the estimated function may be used to predict the range of sensitivity (Figure 2c). Regression parameters *x*_0_ and d*_x_* were estimated as 19.342 ± 0.244 and 11.631 ± 0.238 (mean ± SE), respectively. When the mean coverage depth exceeds 50-fold, sensitivity levels should be larger than 0.80, and when the mean coverage depth exceeds 100-fold, the sensitivity should increase to 0.90.

The SNPs were determined individually. In total, 338 SNPs were found in the four fragments at a frequency of 17 SNPs per kilo base pair. Of these SNPs, 17 were found in coding regions, with 13 being non-synonymous sites (Table 2).

**Table 2:** Nucleotide polymorphisms in the PRP-PACAPa, PRP-PACAPb, GHRH and GHRHR genes.

Region		Number (bp)	Number of Polymorphic Sites	Nucleotide Diversity		Tajima’s D ^c^	Fu’s Fs		dN/dS ^d^	
				π	θ_w_		Fs	*p*	Stat	*p*
GHRH (KR269823.1)	Synonymous	101.66	2	0.00165	0.00310	−0.59938				
	Silent ^b^	4431.66	112	0.00362	0.00399	−0.27736				
	Non-synonymous	324.34	5	0.00210	0.00243	−0.24643				
	Total gene ^a^	4756	117	0.00352	0.00388	−0.28353	−126.856	0.000	0.250	0.803
PRP-PACAPa (KR269822.1)	Synonymous	127.00	0	0.00000	0.00000	NA				
	Silent ^b^	4457.00	81	0.00318	0.00287	0.00267				
	Non-synonymous	395.00	1	0.00006	0.00040	0.00267				
	Total gene ^a^	4852	82	0.00292	0.00267	0.00267	−134.514	0.000	1.029	0.305
PRP-PACAPb (KR269834.1)	Synonymous	134.82	0	0.00000	0.00000	NA				
	Silent ^b^	5084.82	53	0.00107	0.00164	−1.01201				
	Non-synonymous	465.18	1	0.00016	0.00034	−0.50730				
	Total gene ^a^	5550	54	0.00099	0.00154	−1.02345	−73.275	0.000	0.000	1.000
GHRHR (KR269832.1)	Synonymous	82.19	2	0.00266	0.00384	−0.39137				
	Silent ^b^	4009.19	79	0.00285	0.00311	−0.24244				
	Non-synonymous	265.81	6	0.00311	0.00356	−0.24054				
	Total gene ^a^	4280	85	0.00287	0.00313	−0.25251	−46.197	0.000	0.202	0.840

^a^ The total number of fragment sites was assessed; ^b^ Silent substitutions were considered in coding and noncoding regions; ^c^ All Tajima’s D values were not significant (*p* > 0.10); ^d^ The ratio of the rates of non-synonymous and synonymous substitution.

### 2.2. Nucleotide Diversity and Linkage Disequilibrium

Nucleotide diversity levels (θ_w_) in the four fragments were found to be relatively low, ranging from 0.00154 to 0.00388. The diversity of non-synonymous nucleotide substitutions (θ_w_) was found to be comparable to that of synonymous nucleotide substitutions in the four fragments. Although Tajima’s D value was found to be negative with the exception of PRP-PACAPa, none of the values were found to be significant. *Z*-tests showed an absence of selective pressure on the four fragments. Fu’s Fs statistic showed significant signals in all four fragments, revealing a pattern of haplotype diversity that deviated from the expectation under neutral conditions.

**Figure 3:**
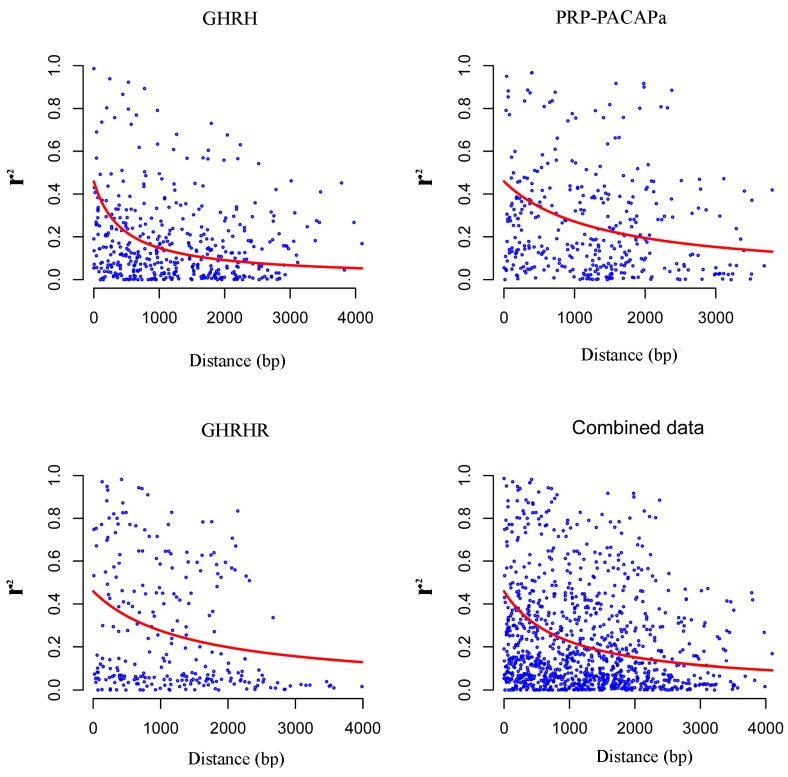
Illustration of the delay in LD for fragments of GHRH, PRP-PACAPa, GHRHR and for the combined data. *r*^2^ points with distance are shown, and delay trends are fit to a mutation-recombination drift model.

LD was preferably estimated using high-frequency polymorphisms, and so we restricted the analysis to SNPs with minor allele frequency (MAF) values of at least 10%. Twenty-seven, one, 30 and 23 SNPs remained for PRP-PACAPa, PRP-PACAPb, GHRH and GHRHR, respectively, and the PRP-PACAPb fragment was excluded. Average values of *r*^2^ for the three GHRH, PRP-PACAPa and GHRHR fragments were recorded at 0.175, 0.258, and 0.272, respectively. Delay curves were plotted for the three fragments and for the combined data (Figure 3). “Half-length” values of LD for GHRH, PRP-PACAPa, and GHRHR and the combined data were recorded at 460, 1465, 1527 and 963 bp, respectively.

### 2.3. The Relationship between Growth Hormone-Releasing Hormone (GHRH), PACAP-Related Peptide/Pituitary Adenylate Cyclase Activating Polypeptide (PRP-PACAP), Growth Hormone-Releasing Hormone (GHRHR) and Growth Traits

Following extraction, the number of common SNPs was found to be 128. The change in estimated data log probability levels (Ln P(D))) between successive *K* values was largest when the number of subpopulations (*K*) was two. Moreover, once the presumed subpopulation quantity reached a value of two, Ln P(D) reached a plateau and increased slightly. Thus the presumed number of subpopulations was set to two. An association was performed, and two markers (KR269823.1:g.475A>C and KR269823.1:g.2143T>C) in the GHRH gene were found to be significantly associated with growth traits (Table 3). The KR269823.1:g.475A>C marker was found in the third position of the first exon’s 24th genetic codon, and the mutation did not cause amino acid changes. This marker was associated with the following five traits: standard length (SL), max body depth (MBD), body weight (BWT), caudal peduncle depth (CPD), and total length (TL), accounting for 16.1%, 12.4%, 17.0%, 9.8% and 13.3% of the total variation, respectively. The KR269823.1:g.2143T>C marker was found in the second intron and was found to be associated with two traits (SL and BWT), which were found to account for 9.2% and 9.0% of the total variation, respectively. Both markers were found to be associated with SL and BWT. Modes of gene action were quantified using the ratio of dominance (d) to additive (a) effects (Table 3). Two marker–trait associations were found to be consistent with partially to fully dominant effects (0.50 < |d/a| < 1.25). The remaining five associations were found to be consistent with additive effects (|d/a| ≤ 0.5).

A haplotype-based association analysis was employed to confirm associations between the identified SNPs in the mixed linear model. A block containing the KR269823.1:g.2143T>C marker and three SNPs (KR269823.1:g.2160A>G, KR269823.1:g.2179C>T and KR269823.1:g.2256G>A) in the GHRH gene was found to be associated with BL (*p* = 0.0389) and BWT (*p* = 0.0294) (Table 4). Three common haplotypes (frequency of >1%) were contained in the block. Proportions of phenotypic variation explained by these haplotypes were recorded at 8.96% and 12.34%, respectively.

**Table 3:** Summary of molecular markers identified via association mapping.

Marker	Gene	Mutation Type	Location	Site	MAF	Trait	*p*	*Q*	*R*^2^ (%)	2a ^1^	d ^2^	d/a ^1,2^
KR269823.1:g.475A>C	GHRH	Synonymous	Exon1	+72	0.24	SL	1.41 × 10^−6^	0.0002	16.1	3.4989	−0.8404	−0.4804
						MBD	3.73 × 10^−5^	0.0048	12.4	0.8379	−0.2173	−0.5186
						BWT	6.10 × 10^−7^	0.0001	17.0	54.7356	−12.8061	−0.4679
						CPD	3.65 × 10^−4^	0.0467	9.8	0.2052	−0.0199	−0.1938
						TL	1.76 × 10^−5^	0.0023	13.3	3.8517	−0.9712	−0.5043
KR269823.1:g.2143T>C	GHRH	Untranslated	Intron2	+1740	0.14	SL	6.34 × 10^−4^	0.0406	9.2	1.6475	−0.1210	−0.1469
						BWT	7.44 × 10^−4^	0.0476	9.0	17.9322	3.5977	0.4013

The *p* value, the significance level for the association (with a significance level of *p* ≤ 0.05), the *Q* value, a correction for multiple testing (false discovery rate FDR(Q) ≤ 0.05), the *R*^2^ value, the percentage of the phenotypic variance explained; MAF, minor allele frequency; SL, standard length; MBD, max body depth; CPD, caudal peduncle depth; TL, total length; ^1^ 2a = |G_BB_ − G_bb_|, where G_ij_ is the trait mean of the ijth genotypic class; ^2^ d = G_Bb_ − 0.5 (G_BB_ + G_bb_), where G_ij_ is the trait mean of the ijth genotypic class.

**Table 4:** Haplotypes significantly associated with growth patterns. Around the detected two associated markers, a block was found to be associated with SL and BWT via a haplotype-based method. The KR269823.1:g.2143T>C marker is denoted as SNP15. The block contained the other three SNPs in the downstream of the gene (KR269823.1:g.2160A>G, KR269823.1:g.2179C>T and KR269823.1:g.2256G>A), which are denoted as SNP16, SNP17 and SNP18, respectively. The block covered 114 bp.

Marker	Trait	*p* Value	*R*^2^	Haplotype	Frequency
KR269823.1:g.2143T>C	SL	0.0389	8.96%	SNPs15–18	
	BWT	0.0294	12.34%	T_A_C_G	0.780
				T_G_T_A	0.070
				C_A_C_G	0.128

*R*^2^, percentage of the phenotypic variance explained; SL, standard length; BWT, body weight.

## 3. Discussion

### 3.1. Target Sequencing

New sequencing methods allow us to process hundreds and thousands of DNA sequences at low costs for each base. However, it is still not feasible to sequence numerous genomes in their entirety due to cost, time constraints, and infrastructure and data storage requirements, especially for non-model genomes. Conversely, linkage and linkage disequilibrium mapping finally focus on sequences of hundreds of thousands to several million base pairs in length, and follow-up fine mapping aims to focus on limited functional genes. Therefore, candidate gene strategies serve as an alternative means for obtaining related markers in non-model species (e.g., the orange-spotted grouper).

PCR-based capturing methods are highly sensitive, consistent and require limited equipment [28]. In this study, four fragments were selected to assess the performance of LR-PCR enrichment approach when applied to the Ion torrent PGM platform. Variance inequality levels in individuals and fragments may reflect the inaccuracy in the process of library preparation and sequencing. The maximum sequencing output for a single individual was found to be 11.2 times that of the minimum one, and this may be attributed to various levels of barcode performance [29], differences between sequencing patches, the amount of DNA loaded for each individual for sequencing and contamination levels. Variance within each fragment is considered mainly attributable to NanoDrop spectrophotometer inconsistencies [30], and variance within each individual is mainly attributed to differing contamination levels, considering more accurate measurement methods employed in standard library construction and sequencing. Overall, the sensitivity of the single amplicon was almost fully determined by the mean coverage depth, and that of numerous amplicons was mainly influenced by variance in the amount of data based on different fragments and less by the specific individuals.

### 3.2. Nucleotide Diversity and Linkage Disequilibrium

Nucleotide variation serves as the basis of selective breeding, and in this study, nucleotide diversity levels were examined for the four candidate fragments. Nucleotide diversity (π) levels ranged from 0.00099 to 0.00352, and non-synonymous site mutations were far less common than those in the silent site. As reported, the diversity of cultured stock would decrease compared with wild stock [31]. Samples examined in the study were full-sib or half-sib with a limited number of parents, thus constituting a typical model of inbreeding. Thus, the nucleotide diversity of the wild population should be larger than the estimated value in this study. Though the neutral test results based on Tajima’s D were not found to be significant, D values were found to be negative in three of four fragments, indicating existence of more low frequency haplotypes. In addition, a neutral state was confirmed through a *Z*-test, and negative values may be attributable to demographic history characteristics, e.g., population expansion. Rather, the number of samples was clearly larger than the number of parents, and the population significantly expanded as a result. However, Fu’s Fs method is particularly sensitive to demographic effects [32], and Fu’s Fs statistic for the four fragments was found to be negative and significant. Therefore, we may infer that the negative Tajima’s D value found is mainly attributable to population growth. LD extension levels declined rapidly, and the half-length became less than 2000 bp. We must note that the LD level was up-biased. The population structure was detected using a Bayesian algorithm. Together, population structure and inbreeding features would inflate LD levels [33]. Thus, a rough estimate was used to provide an indication for the survey of the whole genome level of LD.

### 3.3. Association Mapping of Growth

The mixed linear model has been used for association mapping, which makes it more robust in breeding and the random population. A common concern regarding association mapping pertains to the fact that population structures may trigger false-positive results if not correctly controlled. To control for this risk, population structures must be taken into consideration. However, if a functional allele is correlated with a population structure, population structure control may generate a false negative, particularly for a small sample [34]. Thus, mixed linear models and haplotype-based association mapping methods were employed to detect associations. Although two SNPs (KR269823.1:g.475A>C and KR269823.1:g.2143T>C) in GHRH were found to be significantly associated with growth, the haplotype-based association mapping results repeated this association in only one SNP site (KR269823.1:g.2143T>C) located in the second intron. A possible explanation would be existence of regulatory element on the position [35]. Actually, even though the association methods had identified a SNP significantly, the number of individuals used was rather small and the pedigree was also not clear. All these factors can influence the reliability of the result. So the relation between the SNP and the phenotypes should be confirmed in other independent experiments.

## 4. Experimental Section

### 4.1. The Sample and Phenotype Records

An exhaustive description of the sample has already been presented [4]. In brief, 12 females and 29 males from the first generation of wild orange-spotted grouper captured from the South China Sea near Hainan Island were selected to produce a mass cross population. On 10 December 2010, approximately one month after hatching, over 15,000 fry were collected to rear in the same net cage in the fishery area of Haitang Bay in Sanya, Hainan Island. The experimental stock was fed according to management practices of the fishery. On 8 August 2011, the fish were randomly collected, and growth traits were recorded carefully following methods described in Fishbase [36]. Collected data included body weights (BWT), max body depths (MBD), total lengths (TL), standard lengths (SL), head lengths (HL), body widths (BW), caudal peduncle lengths (CPL), caudal peduncle depths (CPD), snout lengths (SNL), eyeball diameters (ED), and interorbital distances (ID). Condition factor (*K*) was calculated using the following formula: *K* = 100 × BWT/SL^3^. Tissues were cut from the caudal fin of each individual and were preserved in 95% ethanol at −20 °C immediately thereafter. Analysis of variance (ANOVA), normal distribution fit, and correlation tests on phenotypic traits was performed using the IBM SPSS Statistics program (Version 19, IBM SPSS, Chicago, IL, USA). Total DNA were extracted according to a modified proteinase K/phenol extraction protocol [37]. DNA quality and quantity were determined using 1% agarose gels and a UV spectrophotometer (Nanodrop2000/2000c, Thermo Scientific, Pittsburgh, PA, USA).

### 4.2. Candidate Gene Sequencing and SNP Determination

GHRH, PRP-PACAPa, PRP-PACAPb and GHRHR (ID: KR269823.1, KR269822.1, KR269834.1 and KR269832.1, respectively) genes were selected as candidate genes. DNA sequences were retrieved from the genomic sequence of the orange-spotted grouper (unpublished). mRNA sequences were collected from the NCBI database but not for the PRP-PACAPa gene. The mRNA of PRP-PACAPa was predicted in the genomic sequence program of the species and was confirmed through the translated protein. Primer pairs were designed using the Primer Premier 5.0 software program (Premier Biosoft International, Palo Alto, CA, USA) (Table 5). Four pairs of primers were used to amplify DNA from each individual. PCR amplifications were conducted in a final volume of 50 μL containing 10 μL of 5× PrimeSTAR GXL PCR Buffer (Mg^2+^Plus), 4 μL of dNTPs (2.5 mM each), 2 μL of each primer (10 μM), 1 μL of PrimeSTAR GXL DNA Polymerase (1.25 U/μL, TaKaRa, Dalian, China) and 1 μL of genomic DNA (100–500 ng/μL). PCR reactions were performed in an ABI 2720 thermal cycler (Applied Biosystem, Foster City, CA, USA) under the following thermal cycling conditions: denaturation at 95 °C for 3 min; 10 cycles of amplification for 10 s at 98 °C, for 15 s at 63 to 53 °C (with temperatures decreasing by 1 °C for each circle) and for 6.5 min at 68 °C; 25 cycles of amplification for 10 s at 98 °C, 15 s at 53 °C, and 6.5 min at 68 °C with a final extension at 68 °C for 10 min. Production levels were confirmed using 1% agarose gels, and bands of the predicted size were cut and purified using a Universal DNA Purification Kit (Tiangen Biotech (Beijing, China) Co., Ltd.). The quality of purified amplicons was determined using a UV spectrophotometer (Nanodrop2000/2000c, Thermo scientific). Then, amplicons from the same individual were mixed in equimolar amounts. The enriched target sequences of each individual were sequenced on an Ion Torrent Personal Genome Machine platform at Invitrogen Life Technologies. The single-end library of 200 bp was barcoded and constructed according to requisite instructions. The library was sequenced on PGM using chip 318. Raw sequencing read filtering and trimming tasks were performed using Torrent Suit 3.6 with cut-off values and minimum lengths of reads set to Phred quality 18 and 50 bp levels, respectively. Clean read mapping was performed using BWA version 0.7.5 against DNA sequences of the four fragments.

The sensitivity and mean coverage depth of each amplicon varied greatly. Sources of variance from individuals and fragments were examined via a two-way ANOVA. Furthermore, the relationship between sensitivity levels (S) and mean coverage depths (*x*) was fitted to a non-linear regression model based on empirical uses of the Boltzmann function in OriginPro software version 9.0 (OriginLab, Northampton, MA, USA). The Boltzmann function was determined as:

(1) $$\text{S}=-\frac{1}{1+{\text{e}}^{(x-x_{0})/dx}}+1$$

where initial and final sensitivity values are fixed at zero and one, where *x*_0_ is the mean coverage depth at which sensitivity levels reach average values between initial and final sensitivity levels and d*_x_* is a parameter that describes the shape of the curve between upper and lower asymptotes [38].

**Table 5:** Primers of the four genes (four fragments).

ID	Gene	Forward 5′ to 3′	Reverse 5′ to 3′	Amplicon Length ^3^	GenBank Accession Number or Predicted (mRNA)
KR269823.1	GHRH ^1^	TGTGGGAGTGACTGGGAGC	GAGACACGCACAATACCAG	4756	GU966634.1
KR269822.1	PRP-PACAPa ^1^	GATGCACTCCAATGGGAC	GAGTCGTCTGCACAGATG	4852	predicted
KR269834.1	PRP-PACAPb ^1^	GACTGCTCCTTCTTGGTTAA	TTTGTCTCCTGCTCTTCCT	5550	AY869693.1
KR269832.1	GHRHR ^2^	GCTTTGTCGAGCCTGCACC	CAACACAGCAACAACCAGC	4280	GU966635.1

^1^ The amplicon includes the entire CDS; ^2^ The amplicon includes part of the gene; ^3^ The number excludes the length of the two primers at the end of the amplicon.

SNPs for each individual were determined using Samtools version 0.1.19. High-quality SNPs were identified using the following criteria: (1) base quality levels should be greater than or equal to 20 (Phred scale); (2) map quality levels (MAQ) should be greater than or equal to 20 (Phred scale); (3) SNP coverage level should be greater than or equal to 10-fold; (4) SNP quality levels should be greater than or equal to 20; (5) a mutation with a ratio of variant coverage (variant/total) from 10% to 90% should be defined as a heterozygous variant; and (6) INDEL (insertion and deletion) should not be included. Physical coverage (sensitivity) is defined as the percentage of the segment length with a depth of at least 10-fold.

### 4.3. Nucleotide Diversity and Linkage Disequilibrium

The missing genotypes and haplotypes were inferred using PHASE 2.1.1 [39] for each fragment. Varying recombination rates were applied, and burn-in and iteration quantities were set to 100 and 100, respectively. The haplotype probability threshold was set to 0. Nucleotide diversity levels were estimated as π [40] and θ_w_ [41] using DnaSP software version 5.10 [42]. π was defined as the average number of nucleotide differences per site between two sequences, and θ_w_ was defined as the average number of segregating sites per site. Allele frequency spectra were estimated as Tajima’s D. Fu’s Fs were used to detect deviations from neutrality [43]. The number of nonsynonymous substitutions per nonsynonymous site (dN) and the number of synonymous substitutions per synonymous site (dS) were computed using the Nei–Gojobori method [44]. The null hypothesis “H0:dN = dS” was tested using a *Z*-test available through the MEGA program version 6.0 [45].

Linkage disequilibrium (LD) patterns between each pair of detected SNPs in the same fragments were assessed using the Haploview 4.2 program [46]. Squared allele-frequency correlations (*r*^2^) were estimated between sites at an MAF value of greater than 0.1. *r*^2^ Decay with distance was evaluated via nonlinear regression. The expected value of *r*^2^ under a mutation-recombination drift model was determined as:

(2) $$E(r^{2})=[\frac{10+C}{\left(2+C)(11+C\right)}][1+\frac{\left(1+C)(12+12C+C^{2}\right)}{n(2+C)(11+C)}]$$

where *n* is the sample size and where *C* = 4Nc. The nonlinear model contains a single coefficient that is the least-squares estimate for *C* for each bp distance between sites [47]. The “half-length” of LD, as a measure of how far significant LD reaches, was defined as the distance at which mean *r*^2^ levels dropped by 50%.

### 4.4. Associations between the Single-Nucleotide Polymorphisms (SNPs) and Phenotype

The detected SNPs were extracted using the following criterion: (1) MAF > 0.05; (2) the ratio of missing data <20%. Structure association mapping was performed using a mixed linear model (MLM) available through the TASSEL software program version 5.1.0 [48]. Pairwise kinship coefficients and population structures were estimated from 252 random SNPs of restriction-site associated DNA sequencing (unpublished). Each random SNP was located on different scaffolds. Pairwise kinship coefficients were estimated using the SPAGeDi software program version 1.3a [49]. The population structure was inferred using the Structure software program version 2.2 [50]. The presumed number of subpopulations (K) was set to between one and 12. Twenty independent runs for each *K* value were performed using the admixture ancestry model and correlated allele frequency model. For each run, the initial burn-in period was set to 750,000 and was followed by 500,000 MCMC iterations. The most likely *K* value was denoted by Δ*K*, which showed the change in estimated data log probability levels (Ln P(D)) between successive *K* values [51]. Polymorphisms with *Q* values of <0.05 were deemed significantly related sites after SNPs were filtered based on the false discovery ratio [52]. The percentage of phenotypic variation (*R*^2^) explained by each SNP was estimated using the following formula: *R*^2^ = SS_t_/SS_T_ × 100%, where SS_t_ is the variance explained by the marker and where SS_T_ is the total variance. The gene action model was qualified using the ratio of dominance (d) to additive (a) effects [53].

The SNPs around significantly associated sites were combined to determine the block associated with growth. A haplotype-based association analysis was performed via haplotype trend regression (HTR) [54]. Haplotype frequencies were estimated using the expectation maximization (EM) algorithm. An overlapping sliding window used in the four markers approach was employed to test the association, and the significance level was based on a 1000 permutation test.

## 5. Conclusions

In this study, the effects of PRP-PACAPa, PRP-PACAPb, GHRH and GHRHR polymorphisms on phenotype in the important orange-spotted grouper (*Epinephelus coioides*) marine food species were examined. An SNP (KR269823.1:g.2143T>C) located in the second intron of GHRH was found to be significantly related to growth patterns and may be used for breeding purposes. These genes were found to harbor relatively low nucleotide diversity levels and low LD levels in the population.
